# Manipulation of the gut microbiota by the use of prebiotic fibre does not override a genetic predisposition to heart failure

**DOI:** 10.1038/s41598-020-73614-y

**Published:** 2020-10-21

**Authors:** Hamdi A. Jama, April Fiedler, Kirill Tsyganov, Erin Nelson, Duncan Horlock, Michael E. Nakai, Helen Kiriazis, Chad Johnson, Xiao-Jun Du, Charles R. Mackay, Francine Z. Marques, David M. Kaye

**Affiliations:** 1 Heart Failure Research Group, Baker Heart and Diabetes Institute, St Kilda Rd Central, PO Box 6492, Melbourne, VIC 8008 Australia; 2 Hypertension Research Laboratory, School of Biological Sciences, Faculty of Science, Monash University, Melbourne, Australia; 3 Experimental Cardiology Laboratory, Baker Heart and Diabetes Institute, Melbourne, Australia; 4 Monash Micro Imaging, Monash University, Melbourne, Australia; 5 Infection and Immunity Program, Monash Biomedicine Discovery Institute, Monash University, Melbourne, Australia; 6 Department of Biochemistry and Molecular Biology, Monash University, Melbourne, Australia; 7 Department of Cardiology, Alfred Hospital, Melbourne, Australia; 8 Faculty of Medicine Nursing and Health Sciences, Monash University, Melbourne, Australia

**Keywords:** Cardiology, Gastroenterology

## Abstract

Increasing evidence supports a role for the gut microbiota in the development of cardiovascular diseases such as hypertension and its progression to heart failure (HF). Dietary fibre has emerged as a modulator of the gut microbiota, resulting in the release of gut metabolites called short-chain fatty acids (SCFAs), such as acetate. We have shown previously that fibre or acetate can protect against hypertension and heart disease in certain models. HF is also commonly caused by genetic disorders. In this study we investigated whether the intake of fibre or direct supplementation with acetate could attenuate the development of HF in a genetic model of dilated cardiomyopathy (DCM) due to overexpression of the cardiac specific mammalian sterile 20-like kinase (Mst1). Seven-week-old male mice DCM mice and littermate controls (wild-type, C57BL/6) were fed a control diet (with or without supplementation with 200 mM magnesium acetate in drinking water), or a high fibre diet for 7 weeks. We obtained hemodynamic, morphological, flow cytometric and gene expression data. The gut microbiome was characterised by 16S rRNA amplicon sequencing. Fibre intake was associated with a significant shift in the gut microbiome irrespective of mouse genotype. However, neither fibre or supplementation with acetate were able to attenuate cardiac remodelling or cardiomyocyte apoptosis in Mst1 mice. Furthermore, fibre and acetate did not improve echocardiographic or hemodynamic parameters in DCM mice. These data suggest that although fibre modulates the gut microbiome, neither fibre nor acetate can override a strong genetic contribution to the development of heart failure in the Mst1 model.

## Introduction

Heart failure (HF) is a complex, multifactorial disease in which treatments are targeted towards specific aetiologies and phenotypes^1,2^. Many processes contribute to the well-recognized progressive nature of HF including cardiac hypertrophy and myocardial fibrosis. Hypertension remains a common cause of HF, mediated by several contributory factors including mechanical load and activation of pro-inflammatory and pro-fibrotic pathways. Recently, alterations in the gut microbiota have emerged as a common feature and potential contributor to the development of heart failure^3^.

The gut microbiota is readily modifiable by dietary intervention and poses an alternate method for therapeutic intervention. For example, resistant starches (RS) are fermented by commensal bacteria in the large intestine leading to the modulation of the gut microbiota and the release of metabolites such as short-chain fatty acids (SCFAs), which are considered postbiotic^4^. We recently showed that manipulation of the gut microbiota by dietary fibre or by direct administration of 200 mM acetate was able to abrogate the development of progressive cardiac remodelling in an experimental model^5^. SCFAs can reduce local inflammation or be up-taken into the systemic circulation and be distributed to distal organs. RS and its associated postbiotics such as acetate have the potential to elicit cardio-protection in pharmacologically-induced models^3^. Furthermore, supplementation with SCFA and prebiotic fibre increase regulatory T cells, which act to dampen inflammation^6^.

Cardiomyocyte loss due to apoptosis is also a key contributor to the progressive nature of HF^7^. In particular, genetic causes of cardiomyopathy account for a substantial portion of the HF burden and these are marked by high levels of cardiomyocyte apoptosis. Several signalling pathways are responsible for apoptosis, including the gene coding for mammalian sterile 20-like kinase 1 (Mst1), an important mediator of cell death^8,9^. Mst1 has been largely studied in cancer biology, while little is known about its role in the pathophysiology of HF^10–12^. Mice with cardiac-specific overexpression of Mst1 have a phenotype consistent with HF due to dilated cardiomyopathy (DCM)^13^. The role of the gut microbiome or its manipulation in DCM is not known.

In this study, we investigated whether modulation of the gut microbiota using RS and direct treatment with the postbiotic SCFA acetate could ameliorate the development of HF in transgenic Mst1 model of DCM.

## Methods

All animal experiments followed the guidelines from the National Medical and Health Research Council of Australia, with approval from the Alfred Medical Research and Education Precinct Animal Experimentation Ethics Committee (E/1476/2014/B), and the recently published gut microbiota guidelines for cardiovascular studies^14^.

### Animal model

Male transgenic mice overexpressing cardiac-specific Mst1 generated on a C57BL/6 background and their wild-type (WT) littermates (generated by^13^) were housed in the same room and had access to food and water ad libitum during the course of the study (n = 7–8/group). Animals were monitored and weighed regularly throughout the study. To confirm the presence of the transgene, mice were externally genotyped at 4 weeks of age by Transnetyx.

### Dietary intervention

At 6 weeks of age, mice were randomly assigned to diet interventions: control chow diet, high RS diet (referred to as ‘high fibre’, SF11-025, Specialty Feeds) or control chow diet with acetate supplementation (magnesium acetate, Sigma-Aldrich, 200 mM in drinking water in combination with the control diet) for 7 weeks. Both diets had similar amounts of protein, lipids, vitamins, and sodium to potassium ratio. Food and acetate were refreshed twice per week for the duration of the study.

### Functional measurements and morphological analysis

During the final week of the study, mice were housed individually in metabolic cages for 24 h. Food and water intake and urine output were measured. On completion of the study, mice were anaesthetised with isoflurane (3.0–4.5% induction and 1.5–2.0% maintenance during the procedure delivered through a mask) and a 1.4F microtipped transducer (Millar) was placed in the carotid artery to measure left ventricular pressure and arterial blood pressure. Organs were rapidly removed, weighed and snap frozen in liquid nitrogen and stored at − 80 °C until further analyses, or placed in 10% formalin for paraffin wax embedding. Cardiac weight index (mg/g) was calculated from the total heart weight (mg) relative to total body weight (g), and tissue to tibia index was calculated from total tissue weight (mg) relative to tibia length (cm).

### Caecal DNA extraction, library preparation and sequencing

DNA from caecal material was extracted (n = 3–6/group) using DNeasy PowerSoil DNA isolation kit (Qiagen) and V4-V5 region of bacterial 16S ribosomal RNA amplified using previously described PCR methods^15^. Briefly, 20 ng of sample DNA was mixed with Platinum Hot Start PCR master mix (ThermoFisher Scientific), 515F and 926R primers (Bioneer) and amplified in a Veriti Thermal Cycler (ThermoFisher Scientific) in triplicates. The resulting PCR product was assessed for quality and concentration obtained using MultiNa MCE-202 Microchip Electrophoresis system (Shimadzu). 240 ng of amplified product per sample were pooled and purified using the PureLink PCR Purification Kit (ThermoFisher). Samples were sequenced using a Illumina Miseq (2 × 300 bp).

### Microbiome data analysis

16S libraries have been processed using DADA2 and phyloseq R packages to pick amplicon sequence variance (ASV) and to form a table of counts. ASVs have been de novo annotated against Greengene Database (gg_13_8_train_set_97.fa.gz) to the genus level. All libraries went through quality filtering, R1 and R2 merging and chimeras removal steps. The first 10 bases at the 5 prime had been removed from all reads, and R1 and R2 reads had been truncated at the 3 prime end to 245 and 160 bases respectively, prior to merging. The resulting table of counts had a total of 432 distinct ASVs, which was used for all of the downstream statistical analysis. Phylogenetic tree was built in few steps with phangorn R package. Step one was to make multiple sequence alignment and convert it into distance matrix. Multiple sequence alignment was done with AlignSeqs() function from DICEPHER R package. The starting, unrooted, tree was built using Neighbor-Joining, NJ() function, following with maximum likelihood tree using pml() function, finally the tree was optimized with nearest-neighbor interchanges (NNI) rearrangements and the model was GTR. Ordinate function from phyloseq package was used to calculate weighted and unweighted unifrac distances and the data was visualised using plot_ordination function from phyloseq R package. To assess the significance of the separation between diet or groups we used PERMANOVA test on weighted UniFrac distances with default, 999 permutation for pseudo F-distribution. The *P* values were Bonferroni corrected (FWER) for multiple testing. *P* < 0.05 was considered significant. Top 10 most abundant taxa at the genus level had been selected in the control group and abundance was plotted as a barplot using ggplot coloring on genus and faceting on the conditions. For differential taxa occurrence, we used metagenomeSeq R package. To normalise the data we used “cumNormStatFast” and “cumNorm” function and “fitFeatureModel” for fitting linear model.

### Histological analyses

For histological analyses, the top part of the heart was cut into 4 μm thick sections and stained for collagen using standard Masson’s trichrome protocol as previously described^5^. Perivascular and interstitial fibrosis levels were quantified in the heart in 10 random fields per section using an Olympus BH2 microscope (400 × magnification) in a blinded fashion and macros (developed by C.J.) were used to identify the amount of collagen in the FIJI processing package (ImageJ version 1.52p). Collagen levels were expressed as a percentage of the area of the region of interest. For analysis of cell size, 4 μm thick sections were incubated with protein blocking agent and stained with Wheat germ agglutinin-Texas red conjugate. In situ cell death detection kit Fluorescein (Roche) was used to detect TUNEL positive cells on the same sections according to manufacturer protocol. Slides were mounting using ProLong gold antifade mountant with DAPI (Invitrogen). Z-stack images were acquired using a Nikon A1R confocal microscope at 400 × magnification. Analysis were performed blinded using ImageJ software with automated macro (developed by C.J.).

### Flow cytometry

Isolated spleens were dissociated into single cell suspension and lysed with red blood cell lysis buffer. Approximately 1 × 10^6^ cells were stained with a panel consisting of Live/Dead aqua, CD4, CD25, and CD62 antibodies (BD Biosciences). Cells were permeabilised and fixed using manufacturer's guidelines and subsequently stained with Foxp3 (BD Biosciences). Samples were analysed in triplicate using Fortessa LSR flow cytometer (BD Biosciences). Data were processed on FACSDiva software (BD Biosciences).

### Real-time PCR (qPCR)

RNA was extracted from the apical region of heart samples using TRIzol Reagent (Thermo Fisher Scientific) according to manufacturer specifications. RNA was converted to cDNA using the High Capacity cDNA Reverse Transcription kit (Thermo Fisher Scientific). Amplification reactions used the SYBR Green PCR master mix in a QuantStudio qPCR instrument (both from Thermo Fisher Scientific). Samples were run in duplicates. Glyceraldehyde 3-phosphate dehydrogenase (*Gapdh*) gene was used as reference transcript and conditions are listed in Supplementary Table 1. Significance was assessed by 2^−ΔΔCT^ method.

### Statistical analyses

Statistical analysis and graphing were performed on GraphPad Prism (version 8). Normality of data was determined using Shapiro–Wilk’s normality test. Two-way ANOVA (adjusted with post-hoc Benjamini and Hochberg’s false discovery rate for multiple comparisons but not for repeated measures) was used to compare the data between genotypes (wild-type and Mst1) and diet (control, high fibre and acetate). Data are presented as mean ± SEM, and those with a *P* (adjusted for multiple comparisons when there were more than 2 groups) < 0.05 considered significant.

## Results

### Fibre intake significantly modified the gut microbiome independently of genotype

**Figure 1:**
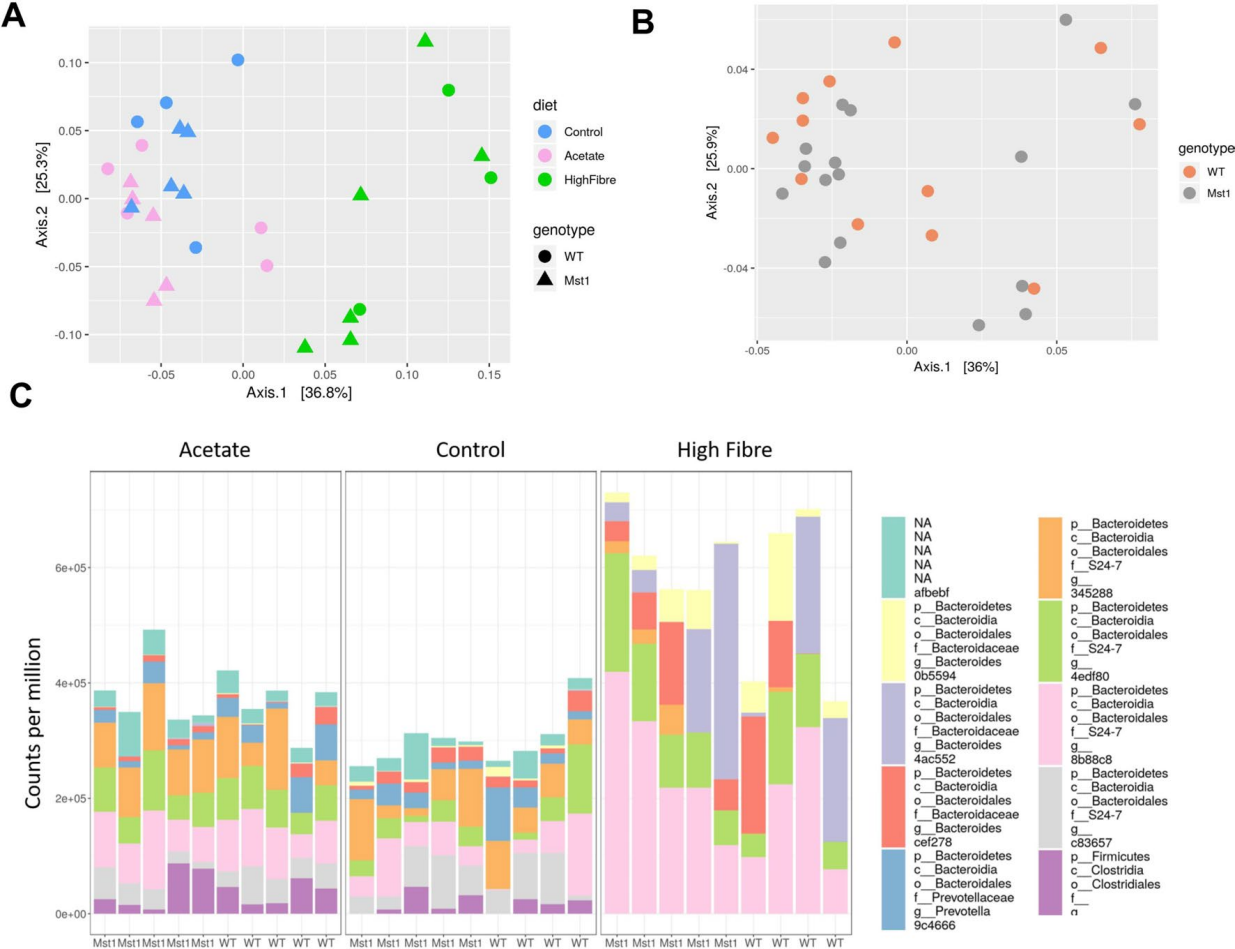
Fibre intake modulated the gut microbiome irrespective of genotype. (**A**) Principle coordinate analysis plot showing weighted unifrac beta diversity sorted according to diet. (**B)** Principle coordinate analysis plot showing beta diversity sorted on genotype. (**C**) Relative abundances of top 10 microbial taxa sorted by diet and acetate supplementation. (n = 3–6/group).

Following a 7-week dietary intervention with high fibre or acetate supplementation, there was a shift in the composition of the gut microbiome of both transgenic Mst1 and WT mice (Fig. 1). We assessed β-diversity using weighted and unweighted uniFrac distances and observed clustering of the samples based on diet within group (Fig. 1A, *P* = 0.003) and not genotype (Fig. 1B). We observed 133 differentially abundant phyla between the gut microbiome of control and high fibre fed mice, but only 19 taxa were different in mice on control diet versus control diet supplemented with acetate mice (Supplementary Fig. 1 and Table 2). For the top ten differential taxa we found that those belonging to the phylum Bacteroidetes were highly enriched in the high fibre samples (Fig. 1C). High fibre diet substantially increased the abundance of Bacteriodales compared to the control diet and acetate supplementation. These findings suggest that diet was the main modulator of the gut microbiome independently of the genotype.

### Fibre and acetate supplementation did not prevent cardiomyopathy in Mst1 mice

**Figure 2:**
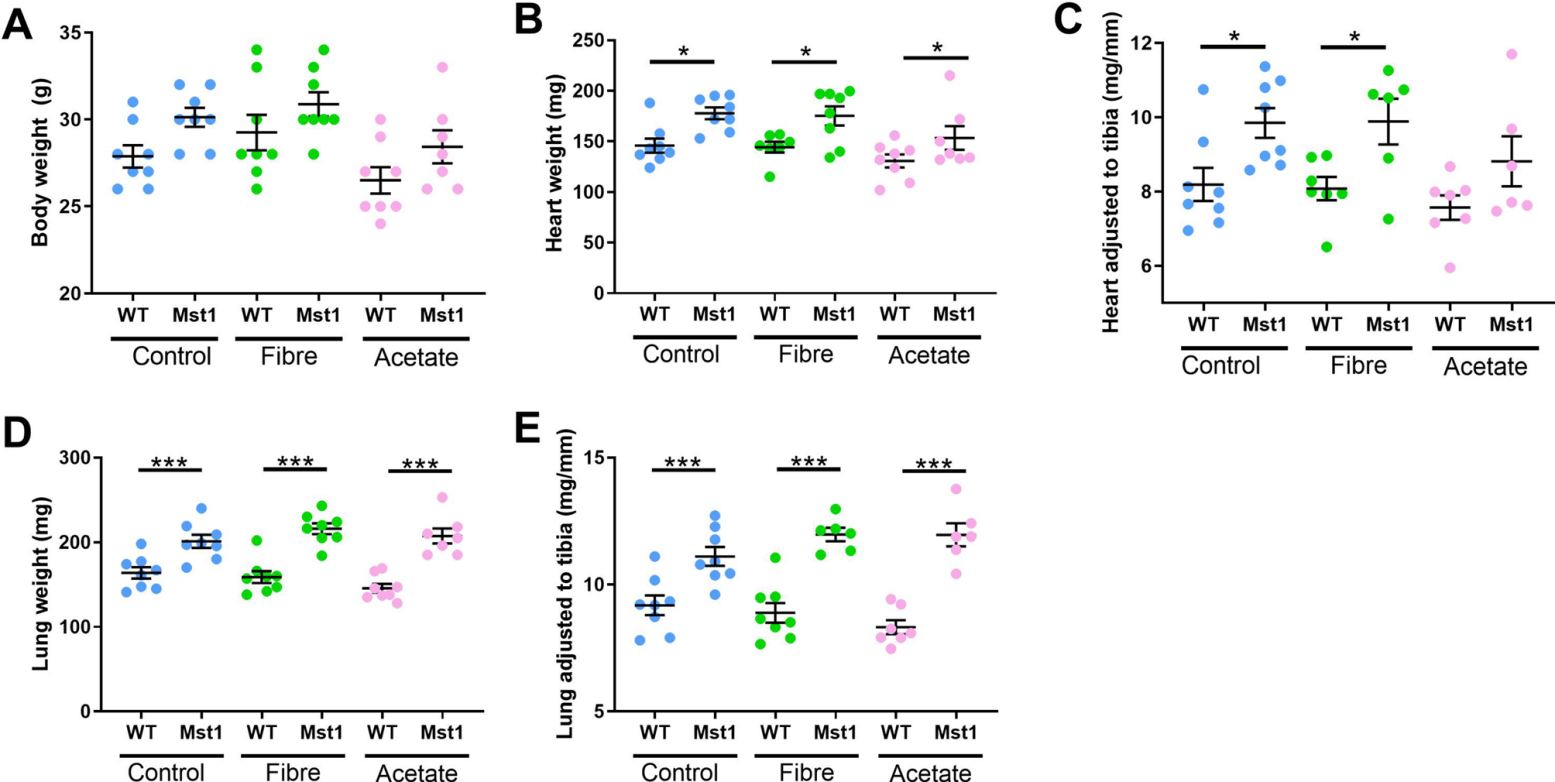
Fiber and acetate supplementation did not prevent the development of cardiac hypertrophy and pulmonary congestion in Mst1 mice. (**A**) Changes in body weight at endpoint of wild-type (WT) and transgenic-Mst1 (Mst1) mice on control diet, high fibre diet and acetate supplementation. (**B**) Heart weight at endpoint of WT and transgenic-Mst1 mice on control diet, high fibre diet and acetate supplementation. (**C**) Heart size normalized to tibia length (mg/mm) of WT and transgenic-Mst1 mice on control diet, high fibre diet and acetate supplementation. (**D**) Lung weight at endpoint of WT and transgenic-Mst1 mice on control diet, high fibre diet and acetate supplementation. (**E**) Lung size normalized to tibia length (mg/mm) of WT and transgenic-Mst1 mice on control diet, high fibre diet and acetate supplementation. Error bar denote mean ± SEM. *P < 0.05, ***< 0.001. n = 7–8/group. 2-way ANOVA used to analyse data.

Mst1 mice had a higher body weight compared to diet-matched WT mice (Fig. 2A). Consistent with previous findings, Mst1 mice had significantly larger heart weight (Fig. 2B, *P* = 0.012), heart to tibia length (Fig. 2C, *P* = 0.022) and heart to body weight ratios (Fig. 2D, q = 0.007). High fibre diet or acetate supplementation did not affect cardiac size (Fig. 2B, C, all *P* > 0.05). Mst1 transgenic mice had significantly larger lung and lung to tibia ratio which was also not prevented by supplementation with acetate or high fibre diet (Fig. 2D, E). Together these data suggest that manipulation of the gut microbiota by intake of RS or direct postbiotic supplementation were unable to protect against the development of cardiac hypertrophy or lung congestion in this severe DCM model.

### Dietary intervention with fibre or acetate did not prevent cardiac dysfunction in Mst1 mice

**Figure 3:**
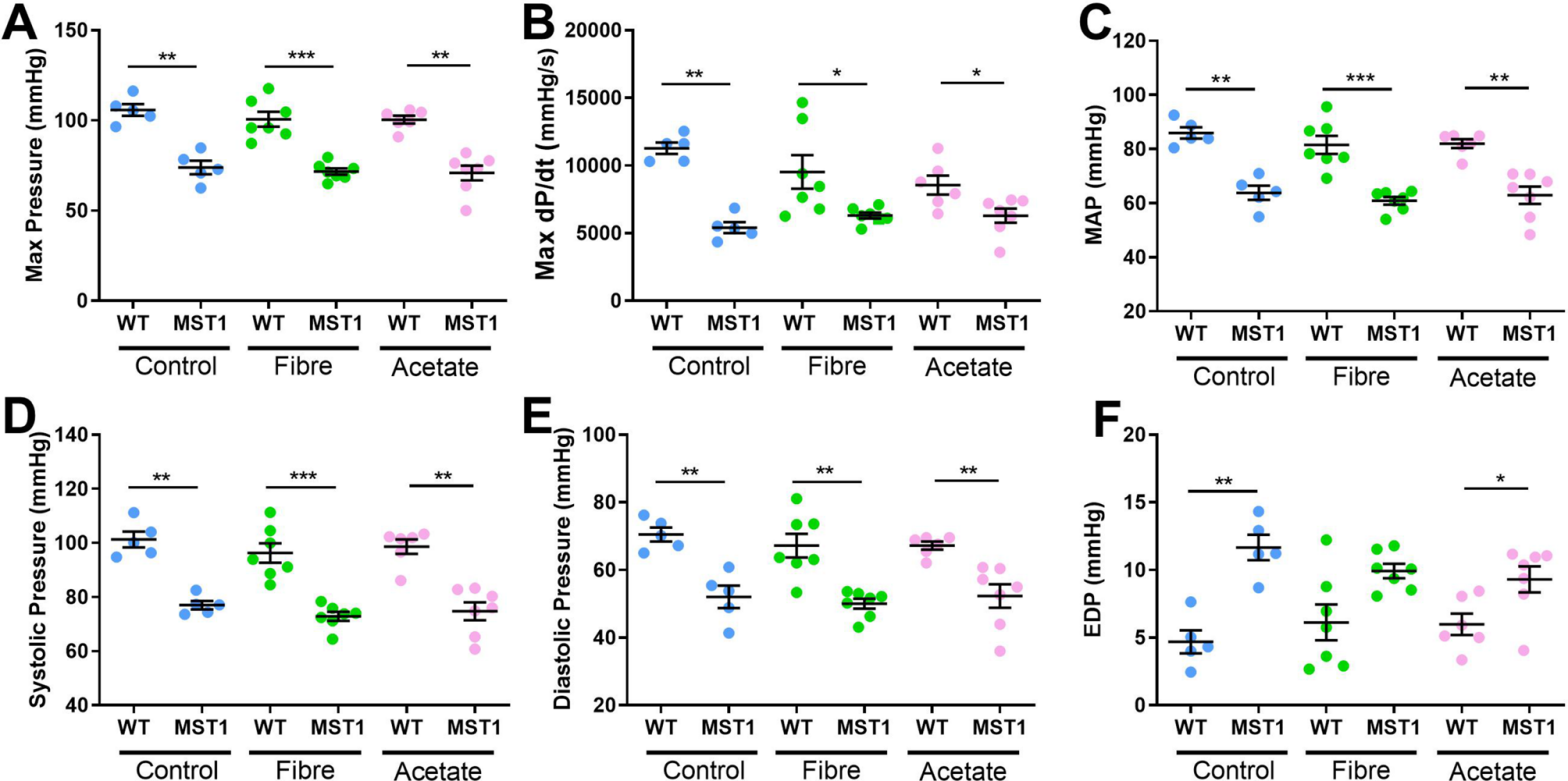
Fiber and acetate supplementation did not rescue cardiac dysfunction in Mst1 mice. Shows cardiac function for wild-type (WT) and transgenic-Mst1 (Mst1) mice on control diet, high fibre diet and acetate supplementation. (**A**) Maximum pressure as measured by cardiac catheter. (**B**) Maximum dP/dt as measured by cardiac catheter. (**C**) Mean arterial pressure as measured by cardiac catheter. (**D**) Systolic pressure as measured by cardiac catheter and (**E**) Diastolic pressure as measured by cardiac catheter and (**F**) End diastolic pressure as measured by cardiac catheter. Error bar shows mean ± SEM. *P < 0.05, **< 0.01 ***< 0.001. n = 5–7/group. 2-way ANOVA used to analyse data.

Mst1 mice had lower maximum pressure (Fig. 3A, *P* < 0.001), maximum dP/dt (Fig. 3B, *P* < 0.001), and blood pressure (Fig. 3C–E, *P* < 0.001), but higher end diastolic pressure (Fig. 3F, *P* < 0.001), compared to wild-type mice, and these measures were not normalised with RS or acetate intake. Together, these data suggest manipulation of the gut microbiota by intake of fibre and acetate supplementation did not reverse cardiac dysfunction in the Mst1 transgenic model.

We then looked into the cross-sectional cardiomyocyte cell size using wheat-germ agglutinin and confocal microscopy. We found that there was no difference in cardiomyocyte area of WT and transgenic Mst1 mice on any of the diets (Supplementary Fig. 2A,B), which suggests that there was no cardiomyocyte hypertrophy. Similar results were found regarding no change in cell death across models and diets (Supplementary Fig. 2C, D).

### Fibre and acetate intake did not prevent cardiac remodelling and fibrosis

**Figure 4:**
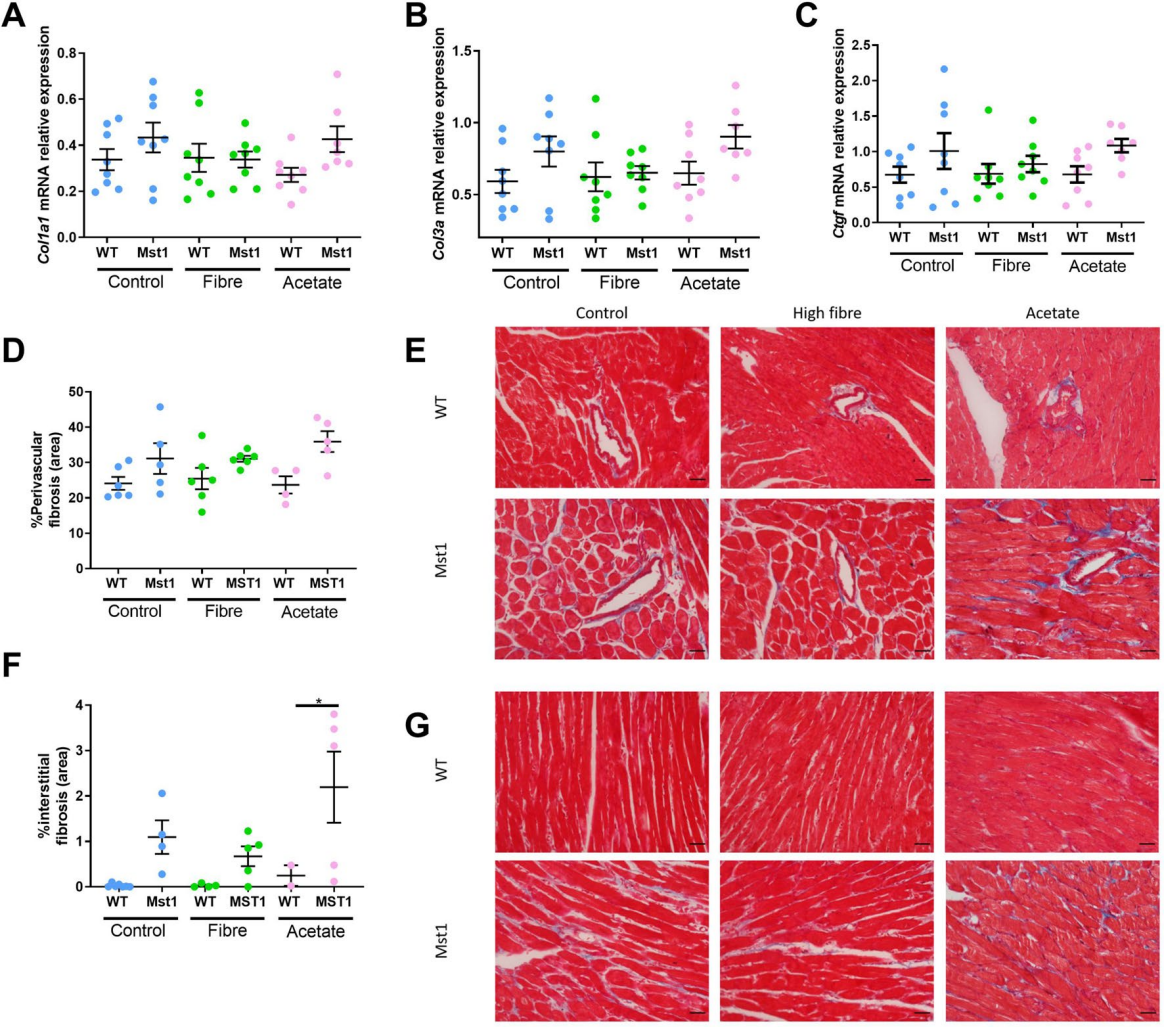
Dietary fiber did not protect against Mst1-overexpression induced cardiac remodeling and fibrosis. (**A**) Shows expression of *Col1a1* mRNA, (**B**) *Col3a1* mRNA, (**C**) *Ctgf* mRNA, all relative to *Gapdh* mRNA with no significant difference found between any of the groups when adjusted for multiple comparisons (FDR q-value < 0.05). (**D)** Quantification of perivascular fibrosis as a percentage of vessel area and (**E**) representative images of perivascular fibrosis captured at 40 × magnification. (**F**) Quantification of interstitial fibrosis as a percentage of total field of view area and (**G**) Representative images of interstitial fibrosis captured at 400 × magnification. Scale bar indicates 100 μm. Error bars shown denote mean ± SEM. *P < 0.05. n = 2–7/group. 2-way ANOVA used to analyse data.

Given that Mst1 transgenic mice exhibit significantly larger hearts when compared to wild-type mice, we wanted to determine whether fibre protected against the accumulation of collagen. We first assessed expression of collagen 1a1 (*Col1a1*) (Fig. 4A), collagen 3a1 (*Col3a1*) (Fig. 4B) and connective tissue growth factor (*Ctgf*) mRNA (Fig. 4C) in the heart by qPCR. Control-fed Mst1 transgenic mice had non-significant but higher levels of *Col1a1, Col3a1* and *Ctgf* mRNA compared to WT mice (*P* = 0.10–0.18). The addition of RS or acetate did not affect the expression of these genes. These findings were consistent with Masson’s trichrome staining (Fig. 4D–G).

### Resistant starches increased the number of splenic T-regulatory cells

**Figure 5:**
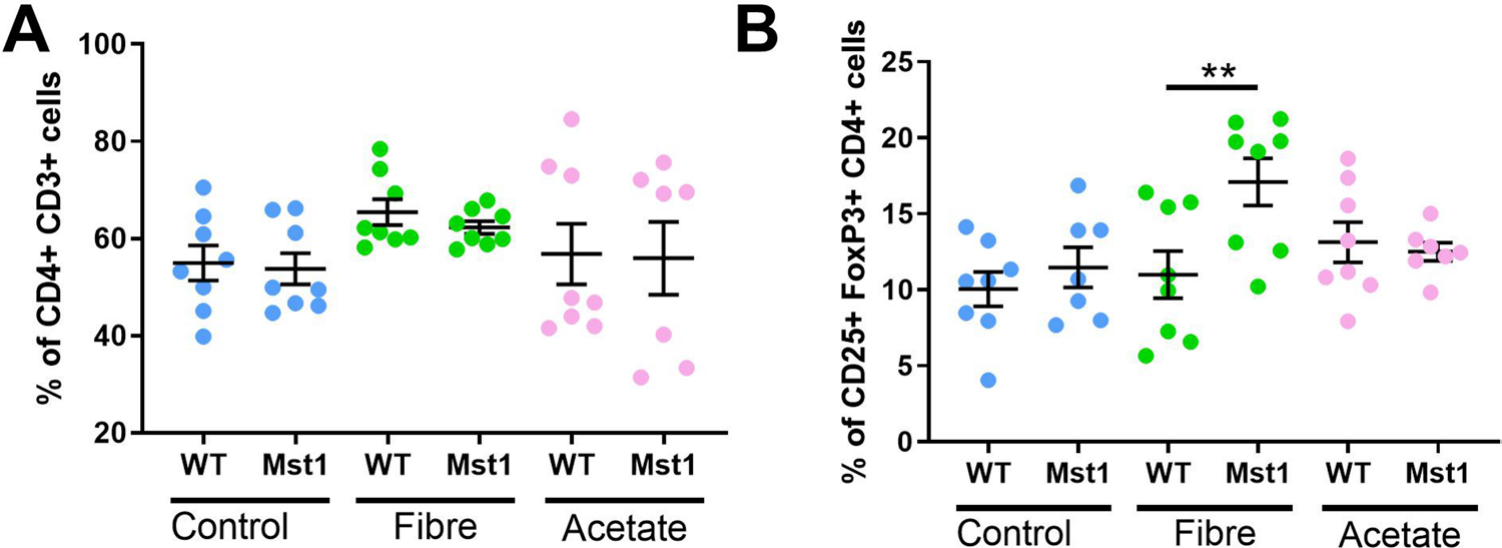
Fiber but not acetate increased splenic T-regulatory cells in Mst1 mice. (**A**) Percentage frequency of CD4+ T-cells as a total of CD3+ T-cells. (**B**) Percentage frequency of CD25+ FoxP3+ T-regulatory cells as a percentage of total CD4+ cells. Error bars show mean ± SEM. **P < 0.01. n = 7–8/group. 2-way ANOVA used to analyse data.

Due to the connection between the gut microbiota, fibre and the immune system^16,17^, we investigated whether RS and acetate changed immune cell populations in the spleen. We found that there was no change in splenic CD4+ (Fig. 5A), however we observed a significant expansion of splenic T regulatory (Treg) cells in Mst1 mice fed a high fibre diet (*P* = 0.009) when compared to wild-type mice on the same diet, and a non-significant increase compared to Mst1 mice on the control diet (*P* = 0.05, Fig. 5B).

### Mst1 and wild-type mice consumed the same amount of food or water

There was no difference in food intake according to diet or supplementation with acetate (Supplementary Fig. 3A). Mst1 mice on a control diet consumed less water when compared to those fed a high fibre diet (Supplementary Fig. 3B, *P* = 0.003), but this was not reflected in urine output (Supplementary Fig. 3C) and was likely part of faecal output. Mice on acetate supplementation consumed significantly less water than mice on the control diet (Supplementary Fig. 3B, *P* = 0.029), likely due to the change in taste. This was reflected in the urine output (Supplementary Fig. 3C, *P* = 0.0311).

## Discussion

Here we assessed the impact of fibre intake (in the form of RS) on the gut microbiota of mice genetically predisposed to developing DCM through constitutive cardiac overexpression of Mst1. We also tested whether the gut metabolite acetate, produced by fermentation of prebiotic fibre by gut commensal bacteria, was able to ameliorate cardiomyopathy in DCM mice compared to WT littermates. We found that, irrespectively of their genotype, a high fibre diet caused a dramatic shift in the composition of the gut microbiome. This is in line with previous studies that have shown the gut modulatory activity of a high fibre intake^5^. However, neither fibre nor acetate prevented cardiac remodelling, hypertrophy and overall dysfunction in DCM animals. While fibre might be used as a therapeutic intervention to reverse gut dysbiosis reported in HF subjects^18^, our findings suggest that, in individuals genetically predisposed to develop cardiomyopathies, fibre may not aid cardiovascular health. Although increased fibre intake has been associated with a decrease in all-cause mortality and, more specifically, cardiovascular mortality^19^, our findings imply that manipulations of the gut microbiota and their metabolites are not able to override a strong genetic predisposition to the development of cardiomyopathy, at least in the Mst1 context. Phenotype of mice with constitutive overexpression of Mst1 are consistent with heart failure and DCM^13^. These mice are described as having enlargement of all four heart chambers, decreased wall thickness, blood clots and hemodynamic defects including increased LVEDP and decreased LV ejection fraction^13,20^.

The role of the gut microbiota in HF is still emerging. There is undoubtedly an intimate link between gut homeostasis and the failing heart. Mechanistically, reduction in blood flow leads to adverse hypoxic state plus venous congestion in the gut which can promote loss of gut barrier integrity, bacterial translocation and systemic inflammation^21^. In support of this, the gut microbiome during HF is different compared to healthy controls^22^. Patients with HF exhibit signs of intestinal dysfunction including morphological changes such as increased gut wall thickness indicating oedema and higher permeability^23^. Moreover, these patients show signs of gut dysbiosis with bacteria growing within the intestinal mucus layer^23^. In support of this, a rat model of HF utilising the coronary artery ligation method showed a shift in the gut microbiome of those that received the surgery compared to those receiving a sham surgery^24^. The degradation of the mucus layer is understood to be perpetuated by a decrease in the consumption of dietary fibre, which is normally used as a microbial fuel source, and promotes the expansion of pathobionts^4,25^. The addition of fibre in the diet would supress growth of these pathogenic bacteria and promote microbial communities that ferment fibre, such as those belonging to the Bacteroidetes phylum. Administration of probiotics has also been reported to improve the cardiac phenotype and normalised the gut microbial composition in the coronary artery ligation rat model^24^. In the present study, we did not observe a difference in the gut microbiome of WT and Mst1 mice, however, intake of fibre modulated the gut microbiota. Despite changing the gut microbial composition with prebiotic fibre, we did observe differences on the cardiac phenotype. This may be due to the relatively early origin of the cardiomyopathy in this model.

In a previous study, we observed that HF and fibrosis were prevented with intake of high dietary fibre or postbiotic treatment in a pharmacologically induced model^5^. This same approach, however, was not sufficient to rescue cardiac dysfunction observed in the DCM model. There are several likely reasons that explain this, one being that this is a severe model of cardiomyopathy. Although we started the interventions at 7 weeks of age, there is evidence that Mst1 transgenic mice have signs of cardiomyopathy as early as 15 days of age^13^. Thus, an even earlier or perhaps in utero intervention could have been more successful to prevent cardiomyopathy in this model. We also observed a modest increase in splenic Treg cells in transgenic Mst1 mice that consumed a diet high in RS, which is consistent with previous studies^26^. Treg cells have been shown to prevent the development of high blood pressure^27^, which is a risk factor for the development of HF. It is unclear, however, if Treg cells are cardioprotective in the setting of HF. Regulatory transcriptional factors, such as FoxP3 and RORyt, and the anti-inflammatory cytokine IL10 are not differentially expressed in the cardiac muscle of chronic HF patients compared to controls^28^. Furthermore, transcriptomic analysis of transgenic Mst1 DCM mice found upregulation of gene sets of interferon α,β,γ-signaling in the Mst1 DCM mice, evidence of inflammatory trend in this model^20^. Thus reducing inflammation would be a reasonable approach to mitigating the severe DCM phenotype.

While we did not interrogate differential gut-derived metabolite expression in the present study, others have shown that a diet high in choline (predominantly found in animal products) increases the production of harmful gut-derived metabolites such as trimethylamine (TMA)^29^. TMA is metabolised by hepatic flavin-containing monooxygenases to trimethylamine N-Oxide (TMAO) and multiple animal and human studies to date have demonstrated a positive correlation between TMAO (and precursor molecules such as choline and L-carnitine) with increased incidence of cardiovascular events^30–32^. Conversely, acetate is a microbial-derived metabolite shown to be cardioprotective in animal models^5^. Given that TMAO is largely a microbial-derived proatherogenic and prothrombotic metabolite, shifting the gut microbiota from TMA-producing to SCFA-producing bacteria will undoubtedly have beneficial effects and possibly abrogate further cardiovascular dysfunction in HF patients.

A limitation of our study is that we only assessed the Treg cells within the spleen, an indicator of systemic inflammation. Patients with DCM have been reported to have deficient and often defective CD4+ T cells^33–35^. Further studies will need to comprehensively investigate the role of other subsets of immune cells, including T cells subsets, within gut and cardiac tissue to delineate their role. How immune cells such as macrophages, which have been shown to have a dual role in the pathogenesis of cardiovascular disease^36^, behave following fibre supplementation is yet to be determined, but our previous findings support that fibre and postbiotic interventions indeed modulate the cardiac transcriptome^5^. While we failed to see abrogation of severe DCM following fibre and acetate supplementation, future studies should measure levels of SCFAs in both plasma and faecal samples. This will undoubtedly reveal differences in the circulating levels of SCFAs which may explain the observed lack of cardio-protection in the DCM model. Furthermore, this study did not account for possible sex differences as we only studied male mice.

In conclusion, although the use of prebiotic (i.e., fibre) and postbiotic (i.e., short-chain fatty acids such as acetate) interventions have been successful in preventing the development of hypertension and HF in pre-clinical models, the current study suggest these interventions may be less successful as treatment for inherited forms of cardiomyopathies. While changes in the gut microbiome could not prevent the severe phenotype observed in the Mst1 transgenic model, we still observed changes in the gut microbiota. This suggests that high fibre intake might still be beneficial to improve gut dysbiosis of HF patients.

## Supplementary information

Supplementary Information.
