# Supplementary material for: Manipulation of the gut microbiota by the use of prebiotic fibre does not override a genetic predisposition to heart failure
Source: Sci Rep. 2020 Oct 21;10:17919. doi: 10.1038/s41598-020-73614-y (PMC7578080; doi:10.1038/s41598-020-73614-y)
Supplement: Supplementary file 1 — Supplementary Information. [file 41598_2020_73614_MOESM1_ESM.docx]

**Online supplement to**

**Manipulation of the gut microbiota by the use of prebiotic fibre does not override a genetic predisposition to heart failure**

Hamdi A. Jama, BSc;^1,2^ April Fiedler, BSc;^1^ Kirill Tsyganov, BSc;^2^ Erin Nelson, MSc;^1^ Duncan Horlock, BSc;^1^ Michael Nakai, BSc;^2^ Helen Kiriazis, PhD;^3^ Chad Johnson, MSc;^4^ Xiao-Jun Du, PhD;^3^ Charles R. Mackay, PhD;^5,6^ Francine Z. Marques, PhD^1,2^*; [David M. Kaye](http://circheartfailure.ahajournals.org/search?author1=David+M.+Kaye&sortspec=date&submit=Submit), MD, PhD^1,7,8^*

*Contributed equally as senior authors

**Corresponding author**: Prof David M. Kaye, MD, PhD, Heart Failure Research Group, Baker Heart and Diabetes Institute, PO Box 6492, St Kilda Rd Central, Melbourne, Victoria 8008, Australia. Phone: +61 3 9076 3263. Fax: +61 3 8532 1100. E-mail: [david.kaye@baker.edu.au](mailto:david.kaye@baker.edu.au)

**Online Supplementary Figures**

**
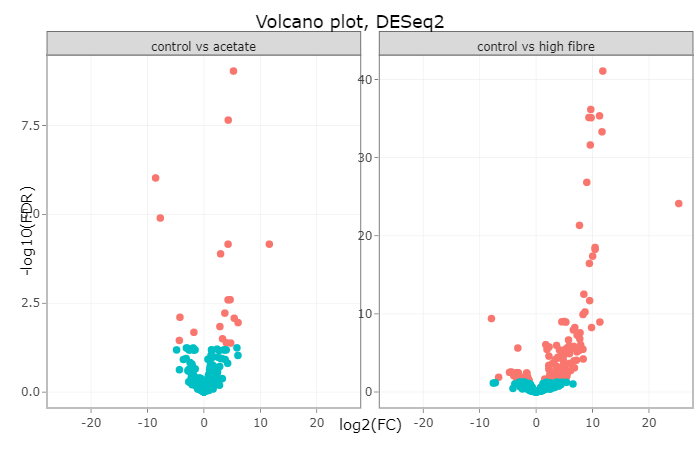
**

**Supplementary Figure 1.** Volcano plot showing the differentially prevalent taxa between control versus acetate (left) and control versus high fibre (right). Red indicates taxa that were significant after adjusted P<0.05


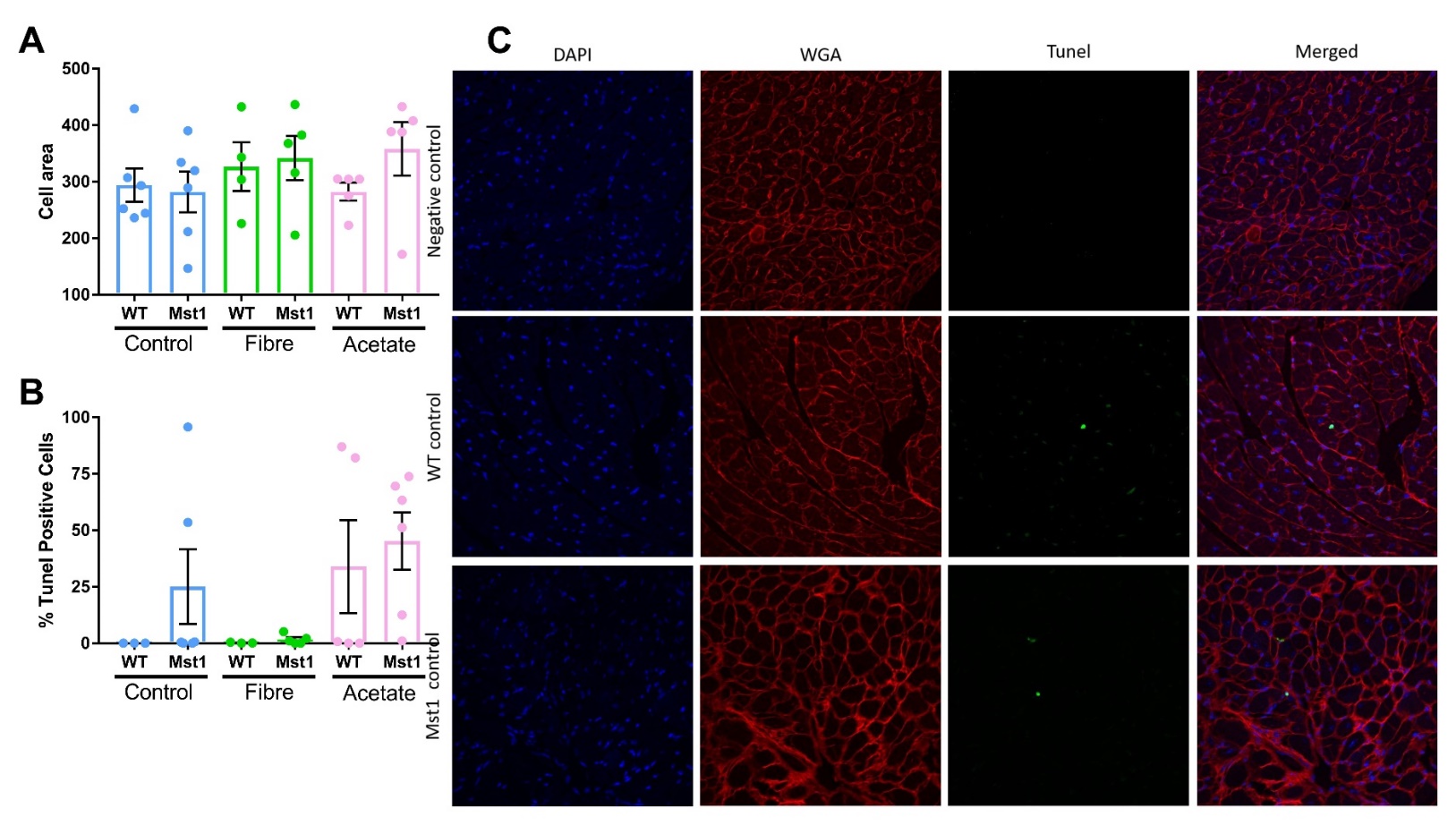


**Supplementary Figure 2. Dietary fibre intake and acetate supplementation had no influence on cell size and apoptosis.**

**A,** Shows quantification of cardiomyocyte cross-sectional cell area as measured by wheat germ agglutinin staining of the cell wall of wild-type (WT) and transgenic-Mst1 (Mst1) hearts on control diet, high fibre diet and acetate supplementation **B,** Shows quantification of apoptotic cells as measured by TUNEL reaction of wild-type (WT) and transgenic-Mst1 (Mst1) hearts on control diet, high fibre diet and acetate supplementation **D,** Representative confocal images showing nuclear Dapi staining (Blue), cell wall stained with WGA (Red) and Tunel positive cells (Green). Magnification 40X. Error bars show mean ±SEM. n=3-6/group. 2-way Anova used to analyse data.

**
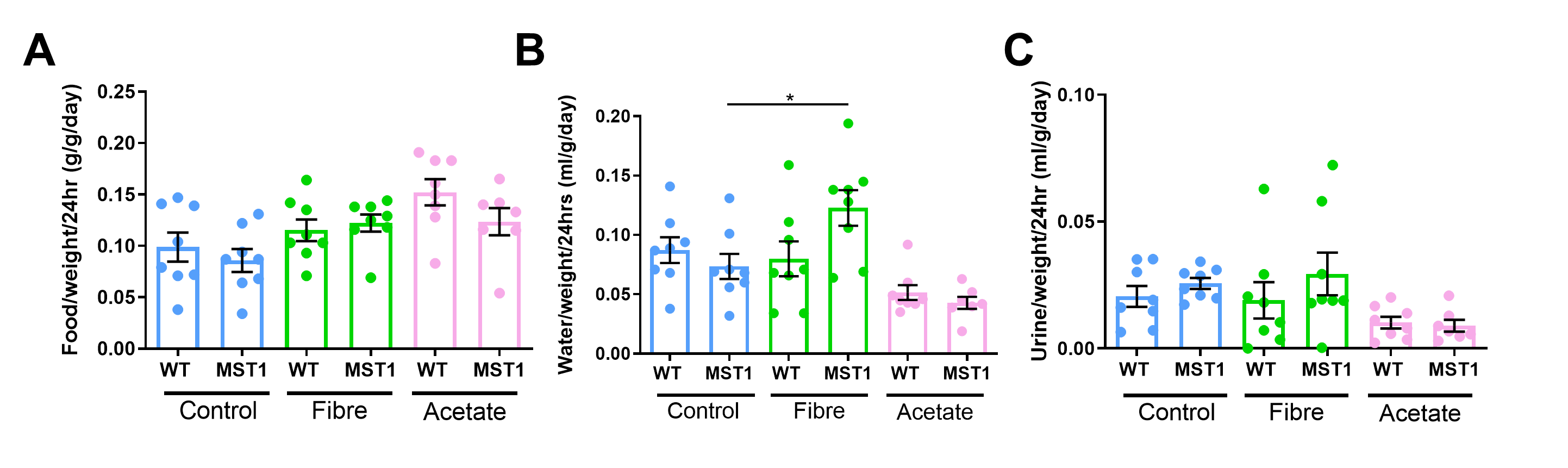
Supplementary Figure 3. Fiber and acetate did not affect food intake or urine output, but they influenced water intake.**

**A,** Shows differences in food consumption over 24 hours normalized to body weight (g/g/day) between wild-type (WT) and transgenic-Mst1 (Mst1) mice on control, high fiber diet and acetate supplementation**. B,** Shows changes water consumption over 24 hours normalized to body weight (ml/g/day) between WT and Mst1 mice on control, high fiber diet and acetate supplementation. **C,** Shows urine output over 24 hours normalized to body weight (ml/g/day) between WT and Mst1 mice on control, high fiber diet and acetate supplementation**.** Error bars shown denote mean±SEM. *P<0.05, **<0.01 ***<0.001. n=7-8/group. 2-way ANOVA used to analyse data.

**Tables**

**Supplementary Table 1.** Primers and conditions used for real-time PCR (qPCR).

| **Official gene symbol** | **Primer Sequence (5’ 🡪 3’)** | **Concentration** | **Annealing temperature** |
| --- | --- | --- | --- |
| *Gapdh* | F: GAGAGTGTTTCCTCGTCCCG | 200nM | 60^o^C |
|  | R: ATGAAGGGGTCGTTGATGGC | 200nM |  |
| *Nppb* | F: CTTCGGTCTCAAGGCAGCA | 200nM | 60^o^C |
|  | R: CAACTTCAGTGCGTTACAGCC | 200nM |  |
| *Ctgf* | F: ACCTGGAGGAAAACATTAAGAAGGG | 200nM | 60^o^C |
|  | R: CACACCCCGCAGAACTTAGC | 200nM |  |
| *Il10* | F: TAATAAGCTCCAAGACCAAGGTG | 200nM | 60^o^C |
|  | R: TCCAGCAGACTCAATACACACT | 200nM |  |
| *Tjp1* | F: GGGCTCCTGGGTTTGGATTT | 200nM | 60^o^C |
|  | R: GCAACTCGGTCATTTTCCTGTA | 200nM |  |
| *Il6* | F: TCGTGGAAATGAGAAAAGAGTTGTG | 200nM | 60^o^C |
|  | R: TCCAGTTTGGTAGCATCCATCAT | 200nM |  |
| *Il17* | F: AGAGCTTCATCTGTGTCTCTGA | 200nM | 60^o^C |
|  | R: TTGGCCTCAGTGTTTGGACA | 200nM |  |
| *Tnfα* | F: ATCGGTCCCCAAGATGA | 200nM | 60^o^C |
|  | R: TGGTGGTTTGTGAGTGTGAGG | 200nM |  |
| *Col3a1* | F: ACACGCAAGGCAATGAGACT | 200nM | 60^o^C |
|  | R: AAGCAAACAGGGCCAATGTC | 200nM |  |

**Supplementary Table 2.** Differential microbial taxa between control, acetate and high fibre diets (adjusted P-value<0.05).

| **Name** | **log2Fold Change** | **Adjusted P-value** | **Up or down** | **Comparison** |
| --- | --- | --- | --- | --- |
| p__Bacteroidetes;c__Bacteroidia;o__Bacteroidales;f__S24-7;g__;09d763 | 4.28 | 0.0001 | up | control vs acetate |
| p__Firmicutes;c__Clostridia;o__Clostridiales;f__;g__;047a78 | 11.59 | 0.0001 | up | control vs acetate |
| p__Bacteroidetes;c__Bacteroidia;o__Bacteroidales;f__Bacteroidaceae;g__Bacteroides;0b5594 | 2.96 | 0.0001 | up | control vs acetate |
| p__Firmicutes;c__Bacilli;o__Lactobacillales;f__Lactobacillaceae;g__Lactobacillus;5fd415 | 4.65 | 0.0025 | up | control vs acetate |
| p__Firmicutes;c__Bacilli;o__Lactobacillales;f__Lactobacillaceae;g__Lactobacillus;07be89 | 4.28 | 0.0025 | up | control vs acetate |
| p__Proteobacteria;c__Deltaproteobacteria;o__Desulfovibrionales;f__Desulfovibrionaceae;g__;dbe56d | 3.71 | 0.0060 | up | control vs acetate |
| p__Bacteroidetes;c__Bacteroidia;o__Bacteroidales;f__S24-7;g__;c6aab5 | -4.25 | 0.0078 | down | control vs acetate |
| p__Firmicutes;c__Erysipelotrichi;o__Erysipelotrichales;f__Erysipelotrichaceae;g__Allobaculum;9dfa04 | 5.38 | 0.0084 | up | control vs acetate |
| p__Firmicutes;c__Erysipelotrichi;o__Erysipelotrichales;f__Erysipelotrichaceae;g__Allobaculum;acba10 | 6.06 | 0.0111 | up | control vs acetate |
| p__Actinobacteria;c__Coriobacteriia;o__Coriobacteriales;f__Coriobacteriaceae;g__;2019c6 | 2.83 | 0.0144 | up | control vs acetate |
| p__Firmicutes;c__Clostridia;o__Clostridiales;f__Ruminococcaceae;g__Oscillospira;1631e6 | -1.78 | 0.0208 | down | control vs acetate |
| p__Bacteroidetes;c__Bacteroidia;o__Bacteroidales;f__S24-7;g__;d00ab5 | 3.30 | 0.0315 | up | control vs acetate |
| p__Firmicutes;c__Clostridia;o__Clostridiales;f__Ruminococcaceae;g__Ruminococcus;adc26a | -4.35 | 0.0352 | down | control vs acetate |
| p__Actinobacteria;c__Actinobacteria;o__Bifidobacteriales;f__Bifidobacteriaceae;g__Bifidobacterium;032b11 | 3.98 | 0.0409 | up | control vs acetate |
| p__Bacteroidetes;c__Bacteroidia;o__Bacteroidales;f__Prevotellaceae;g__Prevotella;670187 | 4.75 | 0.0417 | up | control vs acetate |
| p__Bacteroidetes;c__Bacteroidia;o__Bacteroidales;f__Prevotellaceae;g__Prevotella;baf379 | -8.56 | <0.001 | down | control vs acetate |
| p__Bacteroidetes;c__Bacteroidia;o__Bacteroidales;f__Prevotellaceae;g__Prevotella;16cc35 | -7.73 | <0.001 | down | control vs acetate |
| p__Bacteroidetes;c__Bacteroidia;o__Bacteroidales;f__S24-7;g__;658723 | 4.31 | <0.001 | up | control vs acetate |
| p__Bacteroidetes;c__Bacteroidia;o__Bacteroidales;f__S24-7;g__;b37e62 | 5.24 | <0.001 | up | control vs acetate |
| p__Bacteroidetes;c__Bacteroidia;o__Bacteroidales;f__S24-7;g__;0a62fe | 8.35 | 0.0001 | up | control vs high fibre |
| p__Proteobacteria;NA;NA;NA;NA;74f275 | 4.80 | 0.0001 | up | control vs high fibre |
| p__Bacteroidetes;c__Bacteroidia;o__Bacteroidales;f__S24-7;g__;4c337a | 3.38 | 0.0001 | up | control vs high fibre |
| p__Bacteroidetes;c__Bacteroidia;o__Bacteroidales;f__S24-7;g__;8659c8 | 7.15 | 0.0001 | up | control vs high fibre |
| p__Firmicutes;c__Clostridia;o__Clostridiales;f__;g__;8fddf3 | 6.70 | 0.0001 | up | control vs high fibre |
| p__Firmicutes;c__Bacilli;o__Lactobacillales;f__Lactobacillaceae;g__Lactobacillus;5fb290 | 2.91 | 0.0002 | up | control vs high fibre |
| p__Bacteroidetes;c__Bacteroidia;o__Bacteroidales;f__Rikenellaceae;g__;bca090 | 5.88 | 0.0002 | up | control vs high fibre |
| p__Bacteroidetes;c__Bacteroidia;o__Bacteroidales;f__Bacteroidaceae;g__Bacteroides;940e8d | 4.89 | 0.0002 | up | control vs high fibre |
| p__Firmicutes;c__Clostridia;o__Clostridiales;f__;g__;1eec7a | 6.19 | 0.0003 | up | control vs high fibre |
| p__Bacteroidetes;c__Bacteroidia;o__Bacteroidales;f__Rikenellaceae;g__;79e00b | 2.24 | 0.0004 | up | control vs high fibre |
| p__Firmicutes;c__Clostridia;o__Clostridiales;f__;g__;6fa23e | 3.67 | 0.0005 | up | control vs high fibre |
| p__Bacteroidetes;c__Bacteroidia;o__Bacteroidales;f__Rikenellaceae;g__;17bb86 | 5.07 | 0.0006 | up | control vs high fibre |
| p__Firmicutes;c__Erysipelotrichi;o__Erysipelotrichales;f__Erysipelotrichaceae;g__Allobaculum;acba10 | 6.79 | 0.0008 | up | control vs high fibre |
| p__Firmicutes;c__Clostridia;o__Clostridiales;f__Lachnospiraceae;g__[Ruminococcus];97689e | 4.64 | 0.0010 | up | control vs high fibre |
| p__Firmicutes;c__Clostridia;o__Clostridiales;f__Lachnospiraceae;g__;eeb042 | 2.24 | 0.0011 | up | control vs high fibre |
| p__Actinobacteria;c__Actinobacteria;o__Bifidobacteriales;f__Bifidobacteriaceae;g__Bifidobacterium;6605dc | 3.50 | 0.0013 | up | control vs high fibre |
| p__Firmicutes;c__Clostridia;o__Clostridiales;f__Ruminococcaceae;g__Oscillospira;4b948a | 4.01 | 0.0014 | up | control vs high fibre |
| p__Bacteroidetes;c__Bacteroidia;o__Bacteroidales;f__S24-7;g__;1767e9 | 6.12 | 0.0018 | up | control vs high fibre |
| p__Firmicutes;c__Clostridia;o__Clostridiales;f__Ruminococcaceae;g__Butyricicoccus;0b5d5f | 4.56 | 0.0022 | up | control vs high fibre |
| p__Firmicutes;c__Erysipelotrichi;o__Erysipelotrichales;f__Erysipelotrichaceae;g__Allobaculum;600ac2 | 4.49 | 0.0024 | up | control vs high fibre |
| p__Firmicutes;c__Clostridia;o__Clostridiales;f__[Mogibacteriaceae];g__;f3d946 | 4.91 | 0.0025 | up | control vs high fibre |
| p__Firmicutes;c__Clostridia;o__Clostridiales;f__;g__;d283c4 | -4.43 | 0.0027 | down | control vs high fibre |
| p__Bacteroidetes;c__Bacteroidia;o__Bacteroidales;f__S24-7;g__;adfb19 | 5.50 | 0.0029 | up | control vs high fibre |
| p__Firmicutes;c__Clostridia;o__Clostridiales;f__Lachnospiraceae;NA;d83fdb | 3.51 | 0.0030 | up | control vs high fibre |
| p__Bacteroidetes;c__Bacteroidia;o__Bacteroidales;f__Rikenellaceae;g__;08189a | 2.48 | 0.0031 | up | control vs high fibre |
| p__Bacteroidetes;c__Bacteroidia;o__Bacteroidales;f__Rikenellaceae;g__;3c6193 | 4.61 | 0.0031 | up | control vs high fibre |
| p__Firmicutes;c__Clostridia;o__Clostridiales;f__Lachnospiraceae;g__;2f8f9b | -4.72 | 0.0031 | down | control vs high fibre |
| p__Firmicutes;c__Clostridia;o__Clostridiales;f__;g__;0513a6 | -3.49 | 0.0033 | down | control vs high fibre |
| p__Firmicutes;c__Clostridia;o__Clostridiales;f__Ruminococcaceae;g__Oscillospira;e5f5f7 | 3.89 | 0.0033 | up | control vs high fibre |
| p__Firmicutes;c__Clostridia;o__Clostridiales;f__;g__;ff9fa3 | 5.00 | 0.0033 | up | control vs high fibre |
| p__Firmicutes;c__Clostridia;o__Clostridiales;f__Ruminococcaceae;g__Oscillospira;825257 | 3.72 | 0.0033 | up | control vs high fibre |
| p__Proteobacteria;c__Epsilonproteobacteria;o__Campylobacterales;f__Helicobacteraceae;g__Helicobacter;907d4d | -3.15 | 0.0034 | down | control vs high fibre |
| p__Bacteroidetes;c__Bacteroidia;o__Bacteroidales;f__S24-7;g__;8b88c8 | -1.69 | 0.0038 | down | control vs high fibre |
| p__Bacteroidetes;c__Bacteroidia;o__Bacteroidales;f__Porphyromonadaceae;g__Parabacteroides;e970cc | -1.63 | 0.0038 | down | control vs high fibre |
| p__Firmicutes;c__Clostridia;o__Clostridiales;f__Lachnospiraceae;g__;9408f6 | 5.51 | 0.0045 | up | control vs high fibre |
| p__Firmicutes;c__Clostridia;o__Clostridiales;f__Lachnospiraceae;g__;b5b7b1 | 2.59 | 0.0048 | up | control vs high fibre |
| p__Firmicutes;c__Clostridia;o__Clostridiales;f__Ruminococcaceae;g__Oscillospira;9085ea | 3.51 | 0.0048 | up | control vs high fibre |
| p__Firmicutes;c__Clostridia;o__Clostridiales;f__Lachnospiraceae;g__;acf1e1 | 3.05 | 0.0049 | up | control vs high fibre |
| NA;NA;NA;NA;NA;c865db | 2.60 | 0.0051 | up | control vs high fibre |
| p__Firmicutes;c__Clostridia;o__Clostridiales;f__Ruminococcaceae;g__Ruminococcus;4e9fd3 | 3.76 | 0.0055 | up | control vs high fibre |
| p__Firmicutes;c__Clostridia;o__Clostridiales;NA;NA;9f83c8 | 4.72 | 0.0057 | up | control vs high fibre |
| p__Firmicutes;c__Clostridia;o__Clostridiales;f__Ruminococcaceae;g__Ruminococcus;f74919 | 4.39 | 0.0059 | up | control vs high fibre |
| p__Bacteroidetes;c__Bacteroidia;o__Bacteroidales;f__Rikenellaceae;g__AF12;7c6197 | 4.26 | 0.0060 | up | control vs high fibre |
| p__Proteobacteria;c__Deltaproteobacteria;o__Desulfovibrionales;f__Desulfovibrionaceae;g__;dbe56d | 3.14 | 0.0072 | up | control vs high fibre |
| p__Firmicutes;c__Clostridia;o__Clostridiales;f__Ruminococcaceae;g__Oscillospira;a890ca | 2.88 | 0.0074 | up | control vs high fibre |
| p__Firmicutes;c__Clostridia;o__Clostridiales;f__;g__;d48675 | -3.96 | 0.0082 | down | control vs high fibre |
| NA;NA;NA;NA;NA;223bb4 | 2.38 | 0.0090 | up | control vs high fibre |
| p__Firmicutes;c__Clostridia;o__Clostridiales;f__Ruminococcaceae;g__Butyricicoccus;8f7b7e | 4.47 | 0.0090 | up | control vs high fibre |
| p__Bacteroidetes;c__Bacteroidia;o__Bacteroidales;f__S24-7;g__;e6cc93 | 3.93 | 0.0095 | up | control vs high fibre |
| p__Actinobacteria;c__Actinobacteria;o__Bifidobacteriales;f__Bifidobacteriaceae;g__Bifidobacterium;032b11 | -3.52 | 0.0097 | down | control vs high fibre |
| p__Bacteroidetes;c__Bacteroidia;o__Bacteroidales;f__S24-7;g__;345288 | 2.79 | 0.0097 | up | control vs high fibre |
| p__Firmicutes;c__Clostridia;o__Clostridiales;f__Ruminococcaceae;g__Oscillospira;68f066 | 5.45 | 0.0099 | up | control vs high fibre |
| p__Firmicutes;c__Clostridia;o__Clostridiales;f__Ruminococcaceae;g__Ruminococcus;a36ae3 | 4.03 | 0.0100 | up | control vs high fibre |
| p__Firmicutes;c__Clostridia;o__Clostridiales;f__Ruminococcaceae;g__Ruminococcus;f43079 | 3.08 | 0.0110 | up | control vs high fibre |
| p__Firmicutes;c__Clostridia;o__Clostridiales;f__Ruminococcaceae;g__Oscillospira;156154 | -2.73 | 0.0118 | down | control vs high fibre |
| p__Deferribacteres;c__Deferribacteres;o__Deferribacterales;f__Deferribacteraceae;g__Mucispirillum;9d2775 | -6.64 | 0.0130 | down | control vs high fibre |
| p__Bacteroidetes;c__Bacteroidia;o__Bacteroidales;f__S24-7;g__;9b8bf0 | -1.47 | 0.0134 | down | control vs high fibre |
| p__Bacteroidetes;c__Bacteroidia;o__Bacteroidales;f__Rikenellaceae;g__AF12;7cb5b0 | 3.76 | 0.0140 | up | control vs high fibre |
| p__Firmicutes;c__Clostridia;o__Clostridiales;f__Ruminococcaceae;g__Oscillospira;b5c5f6 | -1.90 | 0.0160 | down | control vs high fibre |
| p__Firmicutes;c__Clostridia;o__Clostridiales;f__;g__;14fe38 | 2.65 | 0.0204 | up | control vs high fibre |
| p__Firmicutes;c__Clostridia;o__Clostridiales;f__Ruminococcaceae;g__Oscillospira;1631e6 | 1.53 | 0.0223 | up | control vs high fibre |
| p__Bacteroidetes;c__Bacteroidia;o__Bacteroidales;f__S24-7;g__;7c3e9e | 3.42 | 0.0235 | up | control vs high fibre |
| p__Firmicutes;c__Clostridia;o__Clostridiales;f__Ruminococcaceae;g__Oscillospira;016be7 | 4.75 | 0.0248 | up | control vs high fibre |
| p__Firmicutes;c__Clostridia;o__Clostridiales;f__Lachnospiraceae;g__Coprococcus;81e6eb | 3.83 | 0.0271 | up | control vs high fibre |
| p__Firmicutes;c__Erysipelotrichi;o__Erysipelotrichales;f__Erysipelotrichaceae;g__Allobaculum;5afe7a | -2.87 | 0.0278 | down | control vs high fibre |
| p__Firmicutes;c__Clostridia;o__Clostridiales;f__Ruminococcaceae;g__Ruminococcus;bcfd03 | -3.20 | 0.0289 | down | control vs high fibre |
| p__Firmicutes;c__Clostridia;o__Clostridiales;f__Ruminococcaceae;NA;888ee8 | 2.12 | 0.0310 | up | control vs high fibre |
| p__Bacteroidetes;c__Bacteroidia;o__Bacteroidales;f__S24-7;g__;64d8b9 | -1.23 | 0.0311 | down | control vs high fibre |
| p__Tenericutes;c__Mollicutes;o__Mycoplasmatales;f__Mycoplasmataceae;g__;a45478 | 1.98 | 0.0319 | up | control vs high fibre |
| p__Bacteroidetes;c__Bacteroidia;o__Bacteroidales;f__S24-7;g__;4edf80 | -1.31 | 0.0399 | down | control vs high fibre |
| p__Bacteroidetes;c__Bacteroidia;o__Bacteroidales;f__Bacteroidaceae;g__Bacteroides;01dbcf | -3.43 | 0.0415 | down | control vs high fibre |
| p__Firmicutes;c__Bacilli;o__Lactobacillales;f__Streptococcaceae;g__Lactococcus;05af07 | -3.20 | 0.0415 | down | control vs high fibre |
| p__Bacteroidetes;c__Bacteroidia;o__Bacteroidales;f__Rikenellaceae;g__;eb4fc5 | 1.60 | 0.0420 | up | control vs high fibre |
| p__Cyanobacteria;c__4C0d-2;o__YS2;f__;g__;e62abe | -2.22 | 0.0466 | down | control vs high fibre |
| p__Firmicutes;c__Clostridia;o__Clostridiales;f__Ruminococcaceae;g__Oscillospira;9930d8 | 1.98 | 0.0470 | up | control vs high fibre |
| p__Firmicutes;c__Clostridia;o__Clostridiales;f__;g__;a9309d | 3.68 | 0.0484 | up | control vs high fibre |
| p__Bacteroidetes;c__Bacteroidia;o__Bacteroidales;f__Bacteroidaceae;g__Bacteroides;f5ef5e | -1.57 | 0.0499 | down | control vs high fibre |
| p__Bacteroidetes;c__Bacteroidia;o__Bacteroidales;f__Bacteroidaceae;g__Bacteroides;4ac552 | -7.91 | <0.001 | down | control vs high fibre |
| p__Bacteroidetes;c__Bacteroidia;o__Bacteroidales;f__Bacteroidaceae;g__Bacteroides;0b5594 | -3.24 | <0.001 | down | control vs high fibre |
| p__Bacteroidetes;c__Bacteroidia;o__Bacteroidales;f__S24-7;g__;9e80a1 | 1.71 | <0.001 | up | control vs high fibre |
| p__Bacteroidetes;c__Bacteroidia;o__Bacteroidales;f__S24-7;g__;5144bc | 1.93 | <0.001 | up | control vs high fibre |
| p__Bacteroidetes;c__Bacteroidia;o__Bacteroidales;f__S24-7;g__;ff2e2f | 2.27 | <0.001 | up | control vs high fibre |
| p__Bacteroidetes;c__Bacteroidia;o__Bacteroidales;f__S24-7;g__;ce75f1 | 2.30 | <0.001 | up | control vs high fibre |
| p__Bacteroidetes;c__Bacteroidia;o__Bacteroidales;f__S24-7;g__;bc6f30 | 3.63 | <0.001 | up | control vs high fibre |
| p__Bacteroidetes;c__Bacteroidia;o__Bacteroidales;f__S24-7;g__;197985 | 3.85 | <0.001 | up | control vs high fibre |
| p__Firmicutes;c__Clostridia;o__Clostridiales;f__Ruminococcaceae;g__Ruminococcus;76a0ae | 4.13 | <0.001 | up | control vs high fibre |
| p__Bacteroidetes;c__Bacteroidia;o__Bacteroidales;f__S24-7;g__;f2428b | 4.29 | <0.001 | up | control vs high fibre |
| p__Proteobacteria;c__Betaproteobacteria;o__Burkholderiales;f__Alcaligenaceae;g__Sutterella;1bb4f9 | 4.53 | <0.001 | up | control vs high fibre |
| p__Bacteroidetes;c__Bacteroidia;o__Bacteroidales;f__S24-7;g__;b49825 | 4.56 | <0.001 | up | control vs high fibre |
| p__Bacteroidetes;c__Bacteroidia;o__Bacteroidales;f__S24-7;g__;889a04 | 5.00 | <0.001 | up | control vs high fibre |
| p__Bacteroidetes;c__Bacteroidia;o__Bacteroidales;f__Bacteroidaceae;g__Bacteroides;ae6a62 | 5.04 | <0.001 | up | control vs high fibre |
| p__Bacteroidetes;c__Bacteroidia;o__Bacteroidales;f__Bacteroidaceae;g__Bacteroides;7af040 | 5.05 | <0.001 | up | control vs high fibre |
| p__Firmicutes;c__Clostridia;o__Clostridiales;f__Lachnospiraceae;g__[Ruminococcus];79c747 | 5.18 | <0.001 | up | control vs high fibre |
| p__Proteobacteria;c__Betaproteobacteria;o__Burkholderiales;f__Alcaligenaceae;g__Sutterella;edece2 | 5.28 | <0.001 | up | control vs high fibre |
| p__Bacteroidetes;c__Bacteroidia;o__Bacteroidales;f__S24-7;g__;722c35 | 5.48 | <0.001 | up | control vs high fibre |
| p__Bacteroidetes;c__Bacteroidia;o__Bacteroidales;f__Bacteroidaceae;g__Bacteroides;4432d6 | 5.49 | <0.001 | up | control vs high fibre |
| p__Bacteroidetes;c__Bacteroidia;o__Bacteroidales;f__Bacteroidaceae;g__Bacteroides;930e4d | 5.75 | <0.001 | up | control vs high fibre |
| p__Firmicutes;c__Clostridia;o__Clostridiales;f__Lachnospiraceae;g__;4498c8 | 5.86 | <0.001 | up | control vs high fibre |
| p__Firmicutes;c__Clostridia;o__Clostridiales;f__Lachnospiraceae;g__;d346fe | 5.92 | <0.001 | up | control vs high fibre |
| p__Firmicutes;c__Clostridia;o__Clostridiales;f__;g__;392394 | 5.92 | <0.001 | up | control vs high fibre |
| p__Firmicutes;c__Clostridia;o__Clostridiales;f__Lachnospiraceae;g__[Ruminococcus];f58704 | 5.94 | <0.001 | up | control vs high fibre |
| p__Firmicutes;c__Clostridia;o__Clostridiales;f__Ruminococcaceae;g__Butyricicoccus;4cc742 | 6.13 | <0.001 | up | control vs high fibre |
| p__Firmicutes;c__Clostridia;o__Clostridiales;f__Lachnospiraceae;g__;2b7308 | 6.32 | <0.001 | up | control vs high fibre |
| p__Bacteroidetes;c__Bacteroidia;o__Bacteroidales;f__S24-7;g__;833f35 | 6.60 | <0.001 | up | control vs high fibre |
| p__Bacteroidetes;c__Bacteroidia;o__Bacteroidales;f__S24-7;g__;2ffb04 | 6.66 | <0.001 | up | control vs high fibre |
| p__Firmicutes;c__Clostridia;o__Clostridiales;f__Ruminococcaceae;g__Oscillospira;bea883 | 6.86 | <0.001 | up | control vs high fibre |
| p__Bacteroidetes;c__Bacteroidia;o__Bacteroidales;f__S24-7;g__;53a38a | 7.23 | <0.001 | up | control vs high fibre |
| p__Firmicutes;c__Bacilli;o__Lactobacillales;f__Lactobacillaceae;g__Lactobacillus;07be89 | 7.26 | <0.001 | up | control vs high fibre |
| p__Firmicutes;c__Clostridia;o__Clostridiales;f__Ruminococcaceae;g__Ruminococcus;8abfe6 | 7.63 | <0.001 | up | control vs high fibre |
| p__Bacteroidetes;c__Bacteroidia;o__Bacteroidales;f__S24-7;g__;bfcf4b | 7.69 | <0.001 | up | control vs high fibre |
| p__Firmicutes;c__Clostridia;o__Clostridiales;f__Lachnospiraceae;NA;7e3e3d | 7.74 | <0.001 | up | control vs high fibre |
| p__Firmicutes;c__Bacilli;o__Lactobacillales;f__Lactobacillaceae;g__Lactobacillus;5fd415 | 7.79 | <0.001 | up | control vs high fibre |
| p__Bacteroidetes;c__Bacteroidia;o__Bacteroidales;f__S24-7;g__;fbf591 | 7.89 | <0.001 | up | control vs high fibre |
| p__Firmicutes;c__Erysipelotrichi;o__Erysipelotrichales;f__Erysipelotrichaceae;g__Allobaculum;9dfa04 | 8.30 | <0.001 | up | control vs high fibre |
| p__Firmicutes;c__Erysipelotrichi;o__Erysipelotrichales;f__Erysipelotrichaceae;g__Allobaculum;744649 | 8.31 | <0.001 | up | control vs high fibre |
| p__Firmicutes;c__Clostridia;o__Clostridiales;f__;g__;1bcc52 | 8.46 | <0.001 | up | control vs high fibre |
| p__Bacteroidetes;c__Bacteroidia;o__Bacteroidales;f__S24-7;g__;cfd369 | 8.67 | <0.001 | up | control vs high fibre |
| p__Bacteroidetes;c__Bacteroidia;o__Bacteroidales;f__S24-7;g__;34d3e3 | 8.98 | <0.001 | up | control vs high fibre |
| p__Bacteroidetes;c__Bacteroidia;o__Bacteroidales;f__Prevotellaceae;g__Prevotella;9c4666 | 9.36 | <0.001 | up | control vs high fibre |
| p__Firmicutes;c__Clostridia;o__Clostridiales;f__Lachnospiraceae;g__;71fe62 | 9.44 | <0.001 | up | control vs high fibre |
| p__Bacteroidetes;c__Bacteroidia;o__Bacteroidales;f__S24-7;g__;d00ab5 | 9.49 | <0.001 | up | control vs high fibre |
| p__Bacteroidetes;c__Bacteroidia;o__Bacteroidales;f__S24-7;g__;f17e99 | 9.60 | <0.001 | up | control vs high fibre |
| p__Bacteroidetes;c__Bacteroidia;o__Bacteroidales;f__S24-7;g__;eb5165 | 9.68 | <0.001 | up | control vs high fibre |
| p__Bacteroidetes;c__Bacteroidia;o__Bacteroidales;f__S24-7;g__;96efcf | 9.75 | <0.001 | up | control vs high fibre |
| p__Bacteroidetes;c__Bacteroidia;o__Bacteroidales;f__Porphyromonadaceae;g__Parabacteroides;9c0518 | 9.83 | <0.001 | up | control vs high fibre |
| p__Bacteroidetes;c__Bacteroidia;o__Bacteroidales;f__S24-7;g__;98217f | 10.02 | <0.001 | up | control vs high fibre |
| p__Firmicutes;c__Clostridia;o__Clostridiales;f__;g__;ffa0fe | 10.45 | <0.001 | up | control vs high fibre |
| p__Bacteroidetes;c__Bacteroidia;o__Bacteroidales;f__S24-7;g__;914890 | 10.46 | <0.001 | up | control vs high fibre |
| p__Bacteroidetes;c__Bacteroidia;o__Bacteroidales;f__S24-7;g__;5f9ffe | 11.24 | <0.001 | up | control vs high fibre |
| p__Bacteroidetes;c__Bacteroidia;o__Bacteroidales;f__Prevotellaceae;g__Prevotella;670187 | 11.30 | <0.001 | up | control vs high fibre |
| NA;NA;NA;NA;NA;afbebf | 11.68 | <0.001 | up | control vs high fibre |
| p__Bacteroidetes;c__Bacteroidia;o__Bacteroidales;f__S24-7;g__;c83657 | 11.82 | <0.001 | up | control vs high fibre |
| p__Tenericutes;c__Mollicutes;o__Anaeroplasmatales;f__Anaeroplasmataceae;g__Anaeroplasma;fc4b95 | 25.28 | <0.001 | up | control vs high fibre |

Legend: p: phylum, c: class, o: order, f: family, g: genus.
